# Prevalence and Diversity of Ly6/uPAR Proteins and Three-Finger LU Domains from the Analysis of Invertebrate Genomes

**DOI:** 10.32607/actanaturae.27898

**Published:** 2026

**Authors:** A. S. Paramonov, Z. O. Shenkarev, E. N. Lyukmanova

**Affiliations:** Shemyakin–Ovchinnikov Institute of Bioorganic Chemistry, Russian Academy of Sciences, Moscow, 117997 Russia; Moscow Center for Advanced Studies, Moscow, 123592 Russia; Faculty of Biology, Shenzhen MSU–BIT University, Guangdong Province, Shenzhen, 518172 China; Faculty of Biology, Lomonosov Moscow State University, Moscow, 119234 Russia

**Keywords:** Ly6/uPAR family, protein evolution, Ctenophora, Placozoa, Porifera, Cnidaria

## Abstract

The family of Three-Finger Proteins (TFPs or Ly6/uPAR) containing three-finger domains (LU domains) with a characteristic three-finger fold was initially characterized for toxins from the venom of snakes of the family Elapidae. TFPs have been described in vertebrates and some invertebrates, where they are known to participate in the regulation of membrane receptors, intercellular recognition, signal transduction, embryogenesis, and the functioning of the nervous, immune, and reproductive systems. Although a vast amount of genomic information has recently become available, the diversity and representation of TFPs in various groups of organisms, as well as the evolution of proteins belonging to this family, remain poorly understood. In this study, we employed the bioinformatics methods to analyze the annotated genomes of various metazoans available in open databases. Protein sequence analysis using hidden Markov models enabled the identification of TFPs in the genomes of the members of all the metazoan phyla, including those most evolutionarily distant from humans: ctenophores (Ctenophora), sponges (Porifera), placozoans (Placozoa), and cnidarians (Cnidaria). Further domain annotation revealed the presence of proteins with three-finger domains and a previously undescribed domain architecture in cnidarians, mollusks, and arthropods. Comparison of the LU domains of early metazoans to those of humans revealed a high degree of sequence similarity (up to 69%) for some proteins. For example, the LU domains of the human activin receptors ACVR and the GPI-anchored proteins Ly6D and Lynx2 (Lypd1) appeared conserved. The function and mechanisms of action of invertebrate TFPs remain unknown. An analysis of the annotated genomes of some fungi and plants and two published genomes of choanoflagellates (Choanoflagellata), a phylum of unicellular organisms that are the most phylogenetically close to the Metazoa, revealed no presence of sequences encoding TFPs. It is hypothesized that TFPs first appeared during the evolution of protozoa, upon the transition of animals to multicellularity.

## INTRODUCTION

Ly6/uPAR family proteins – or **t**hree-**f**inger **p**roteins (TFPs) – contain characteristic three-finger domains, also known as LU domains. These small in size, mostly β-structural domains (consisting of ~ 60–90 amino acid residues) are characterized by a specific folding of the polypeptide chain that includes the domain core (head) stabilized by a system of conserved disulfide bonds and three elongated loops (fingers) projecting outward from it. TFPs typically contain one or several LU domains [1] and can be bound to the membrane via the glycosylphosphatidylinositol (GPI) anchor [2] or an additional C-terminal transmembrane (TM) helix [3]. There also exist secreted Ly6/uPAR proteins, including snake α-neurotoxins [4] and mammalian SLURP proteins [5]. Moreover, LU domains are found within multidomain proteins (e.g., as one of the extracellular domains of activin membrane receptors (ACVRs) and bone morphogenetic protein receptors (BMPRs)). These receptor serine/threonine kinases (RKs) belong to the family of the transforming growth factor β receptor (TGFβR) [6]. Sequences coding for hypothetical G-protein coupled receptors whose extracellular portion also contains the LU domain have recently been confirmed in starfish genomes [7].

It is known that secreted and non-secreted TFPs are present in animals belonging to different phyla and classes. A total of 45 TFP genes, which perform regulatory functions in the immune [8] and reproductive systems [9], as well as the CNS [10], and regulate epithelial tissue formation [5], have been annotated in the human genome. The Lynx2 protein, presumably a regulator of the anxiety level [11], has been detected in the murine CNS, while the avian HEP21 protein plays a crucial role in the development of the reproductive tract [12]. Three-finger toxins from snake venom inhibit nicotinic and muscarinic acetylcholine receptors, acid-sensing ion channels, as well as acetylcholinesterase, and can disrupt the cell membrane integrity [13]. Amphibian TFPs perform a pheromone-like function [14] and are involved in regeneration processes [15]. In fish, TFPs regulate fertilization [16], the innate immune system of the gastrointestinal tract [17], and the Wnt/β-catenin signaling pathway that is crucial for embryogenesis [18]. TFPs regulating sleep [19], the formation of the cell–cell contacts maintaining the blood–brain barrier [20], nociception [21], and the cholinergic system [22] have been discovered in insects. Several TFPs found in echinoderms [7, 23, 24] have been recently reported, but their functions remain unclear.

Current data on the diversity of TFPs are confined to chordates and arthropods, whereas the prevalence of TFPs in other phyla and the evolutionary origin of this protein family remain unexplored. However, recent advances in sequencing technologies (the availability of numerous annotated genomes in public databases) and the possibility to predict the protein spatial structure using tools such as AlphaFold 3 [25] offer extensive opportunities to analyze the presence of TFPs in various organisms. This study presents the results of a search for TFP genes in the genomes of multicellular invertebrates (Metazoa) and the analysis of the identified sequences.

## EXPERIMENTAL

**Datasets**

Sequences deposited in the EnsemblGenomes 61 database [26] were used in this study. Annotated genome assembly datasets from the NCBI GenBank [27] were additionally used for members of the phyla Porifera, Placozoa and Ctenophora, as well as phyla whose genomes are not in EnsemblGenomes [27]. Translated sequences of the proteins, in the form in which they had been deposited in the databases, were analyzed; no additional sequence processing was performed. Species assignments to higher taxa were determined using the NCBI taxonomy database [28] (as of September 5, 2025). The approximate number of described animal species in each phylum is provided according to Catalogue of Life (www.catalogueoflife.org) [29] as of July 2025.

**Searching for LU-domain sequences**

Profile hidden Markov models for TFPs and LU domains belonging to the CL0117 family group (uPAR_Ly6_toxin), a total of 14 profiles, were obtained from the PfamA database, version 38.0 [30]. The profiles were consecutively cross-referenced to the sequence set for each analyzed genome using the hmmsearch tool from the HMMER 3.3.2 software suite [31], with pre-filters disabled (the `--max` parameter). The database size for the calculation of the E-value (threshold 100) was set using the `-Z 81514348` parameter. The domain-level output (`--domtblout`) was analyzed with Python scripts using the BioPython [32] and Biotite [33] libraries. Only the sequences with a LU domain length of at least 60 residues were analyzed.

**Annotation of the identified sequences**

The identified protein sequences containing LU domains were additionally analyzed to search for conserved domains of other types. For this purpose, each sequence was cross-referenced to the PfamA profile database using hmmscan via the pfam_scan.pl software, version 1.6 (J. Mistry, R. Finn; http://ftp.ebi.ac.uk/pub/databases/Pfam/Tools/). Sequences were annotated using the InterProScan 5.76-106.0 software package [34] with the following databases included: AntiFam-8.0, CDD-3.21, Coils-2.2.1, FunFam-4.3.0, Gene3D-4.3.0, Hamap-2025_01, MobiDBLite-4.0, NCBIfam-17.0, PANTHER-19.0, Pfam-37.4, Phobius-1.01, PIRSF-3.10, PIRSR-2025_01, PRINTS-42.0, ProSitePatterns-2025_01, ProSiteProfiles-2025_01, SFLD-4, SignalP_EUK-4.1, SMART-9.0, SUPERFAMILY-1.75, and TMHMM-2.0c. The results were analyzed and filtered using Python scripts. The identified LU domains and domains of other types were manually verified taking into account the data obtained using the InterProScan software package and analyzed using the CDART web-based utility [35]. The presence of a signal peptide, transmembrane helices, and a GPI anchor attachment site were predicted using the SignalP 6.0 [36], DeepTMHMM [37], and PredGPI [38] software, respectively.

In order to eliminate the erroneous sequences arising during sequencing or genome assembly or related to sample contamination, protein sequences with a non-standard domain architecture were verified by a search across the NR database using the BLASTP tool [39]. The presence of corresponding mRNAs was cross-referenced against the TSA (transcriptome shotgun assembly) database using the TBLASTN tool. Searches were restricted to species belonging to a given phylum. If sequences were found in closely related species with an at least 80% overlap with the query sequence and containing the domains of interest, the protein-coding gene was considered genuine. Otherwise, the sequence was discarded as a false result.

The protein spatial structures were predicted on the basis of sequences using the AlphaFold 3 [25] and ESMFold AI tools [40]. The structures were visualized using the MOLMOL molecular graphics program [41].

**Comparison of LU-domain sequences**

The pairwise similarity of manually selected LU-domain sequences was calculated by pairwise global alignment using the Biotite library with a modified BLOSUM62 substitution matrix, the weight of the Cys/Cys substitution set at 99. The proportion of similar residues located at the same positions in the aligned sequences was calculated. Residues were considered similar if the corresponding substitution in the BLOSUM62 matrix had a weight ≥ 0.

## RESULTS AND DISCUSSION

**TFP genes are found in all the phyla of metazoan invertebrates and are presumably absent in plants and fungi**

The representation and diversity of the TFP genes were analyzed across 387 invertebrate genomes belonging to 348 species (*Table 1*). The analyzed genomes encompassed 22 of the 32 metazoan phyla for which annotated genomes had been published at the time of the study (*Fig. 1*). The genes of proteins containing LU domains were detected in all the genomes explored. *Table 1* concisely summarizes the identified TFPs; the comprehensive list of TFP sequences is provided in *Supplementary File 1* (*Supplementary Materials*).

**Fig. 1:**
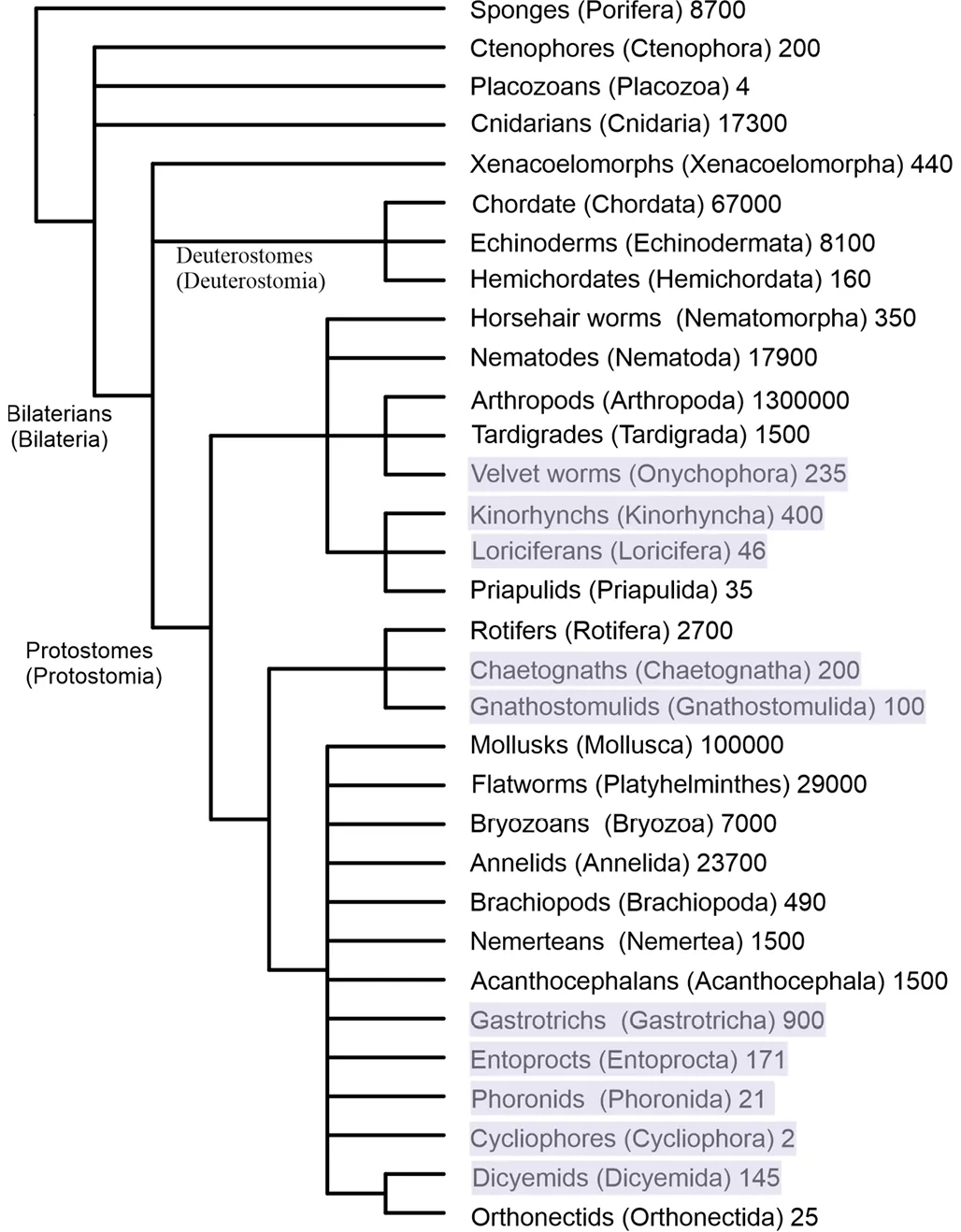
Phylogenetic tree of the phyla of metazoans, constructed from the NCBI taxonomic database [28]. The number of described species is given for each phylum. The phyla whose genomes were not analyzed in the study are highlighted in gray

**Table 1:** Phyla of the multicellular animals (Metazoa) whose genomes were analyzed in this study

Phylum		Number of species described	Number of species analyzed	Number of genomes analyzed	Number of TFP (average per genome)	Domains composition**
Porifera	Sponges	8700	7	8	27 (3)	LU, RK
Ctenophora	Ctenophores	200	2	2	16 (8)	LU, RK, Fig. 2
Placozoa	Placozoans	4	1	1	2 (2)	RK
Cnidaria	Cnidarians	17,300	13	13	563 (43)	LU, RK
Xenacoelomorpha	Xenacoelomorphs	440	3	3	25 (8)	LU, RK
Chordata*	Chordates	67,000	1	1	36 (36)	LU, RK
Echinodermata	Echinoderms	8100	7	7	291 (42)	LU, RK, 7TM
Hemichordata	Hemichordates	160	2	2	110 (55)	LU, RK
Nematomorpha	Horsehair worms	350	1	1	14 (14)	LU, RK
Nematoda	Nematodes	17,900	14	14	599 (43)	LU, RK
Arthropoda	Arthropods	1,300,000	254	290	9066 (31)	LU, RK, 7TM, Fig. 2
Tardigrada	Tardigrades	1500	2	2	217 (109)	LU, RK
Priapulida	Velvet worms	35	1	1	28 (28)	LU, RK
Rotifera	Rotifers	2700	1	1	69 (69)	LU, RK
Mollusca	Mollusks	100,000	24	25	2086 (83)	LU, RK, 7TM, Fig. 2
Platyhelminthes	Flatworms	29,000	4	5	61 (12)	LU, RK
Bryozoa	Bryozoans	7000	2	2	82 (41)	LU, RK
Annelida	Annelids	23,700	5	5	272 (54)	LU, RK
Brachiopoda	Brachiopods	490	1	1	101 (101)	LU, RK
Nemertea	Nemerteans	1500	1	1	58 (58)	LU, RK
Acanthocephala	Acanthocephalans	1500	1	1	3 (3)	LU, RK
Orthonectida	Orthonectids	25	1	1	5 (5)	LU, RK

*The genomes of vertebrates were not analyzed. The phylum Chordata is represented by a single organism

(the lancelet *Branchiostoma lanceolatum*).

**Domain architecture of the detected TFPs. LU – proteins containing exclusively three-finger domains (at least one) and possibly containing a GPI-anchor attachment site or a C-terminal TM helix. RK – membrane receptor kinases containing a LU domain(s) in the N-terminal extracellular region (similar to TGFβR). 7TM – proteins with 7TM helices containing a LU domain(s) in the N-terminal extracellular region (similar to GPCRs). “Fig. 2” – additional non-standard architectures shown in *Fig. 2*.

The number of TFP genes varied significantly across the genomes of organisms belonging to different phyla. The largest number of TFP genes was found in tardigrades (Tardigrada) genomes: (87 in *Hypsibius exemplaris* and 130 in *Paramacrobiotus metropolitanus*), as well as 101 TFPs in the only published genome of a brachiopod (Brachiopoda): *Lingula anatine*. Contrariwise, the smallest number of TFP genes was detected in the genomes of placozoans (Placozoa), obligate parasites (acanthocephalans (Acanthocephala), and orthonectids (Orthonectida)), with only two to six genes per genome (*Table 1*).

Comparative analysis of 1,500 genomes of fungi representing 980 species and 169 genomes of plants representing 158 species was performed. Genes encoding TFPs were detected in neither case. Analysis of the genome of *Olea europaea* (the olive tree) revealed a gene encoding a protein containing the LU domain. The sequence of this domain was the most similar to those in insect TFPs; a search for closely related plant genomes failed to identify any similar genes. Therefore, we surmised that the detected TFP gene could have resulted from sample contamination. Furthermore, two published genomes of choanoflagellates (Choanoflagellata – protists belonging to the group of opisthokonts (Opisthokonta), together with fungi and animals – were analyzed. Choanoflagellates are believed to be the ancestors of Metazoa. No TFPs were detected in two choanoflagellate species (out of ~ 130 species described): *Monosiga brevicollis* and *Salpingoeca rosetta*.

**The TFP genes in the genomes of sponges, placozoans, ctenophores, and cnidarians**

TFPs were previously described in bilaterians (Bilateria), including both protostomes (such as insects) and deuterostomes (chordates (vertebrates) and echinoderms). However, the presence of these proteins in non-bilaterian, the phylogenetically earliest animal phyla, including sponges, placozoans, ctenophores, and cnidarians (*Fig. 1*), is of particular interest, as it is directly related to the question of the evolutionary origin of the three-finger structural motif and TFPs.

We reviewed the genes encoding TFPs in the genomes of sponges, placozoans, and ctenophores, as well as in the genome of the pale anemone *Exaiptasia diaphana* (phylum Cnidaria) (*Supplementary File* 2, *Supplementary Materials*). Domain annotation of the identified TFP sequences (*Fig. 2A*) and the predicted spatial structures (*Fig. 3*) revealed that sponges possess proteins containing one to three LU domains. Almost all these proteins carry signal sequences responsible for secretion; many of them also harbor either the GPI-anchor attachment site or a sequence encoding the TM helix. Therefore, the respective proteins are either secreted or anchored to the extracellular side of the membrane (see examples in *Figs. 2A* (1−3) and *3* (1, 2, 4−9)). Furthermore, genes coding for proteins architecturally similar to TGFβR family receptor kinases carrying the LU domain in their N-terminal ligand-binding portion (see the example in *Figs. 2A* (4) and *3* (3)) were also encountered in sponges. The same set of TFP architectures was also found in the genomes of ctenophores and cnidarians, as well as in the genomes of all the analyzed bilaterians. However, the genome of the placozoan *Trichoplax* was found to contain only two TGFβR-like genes (*Fig. 3* (3)).

**Fig. 2:**
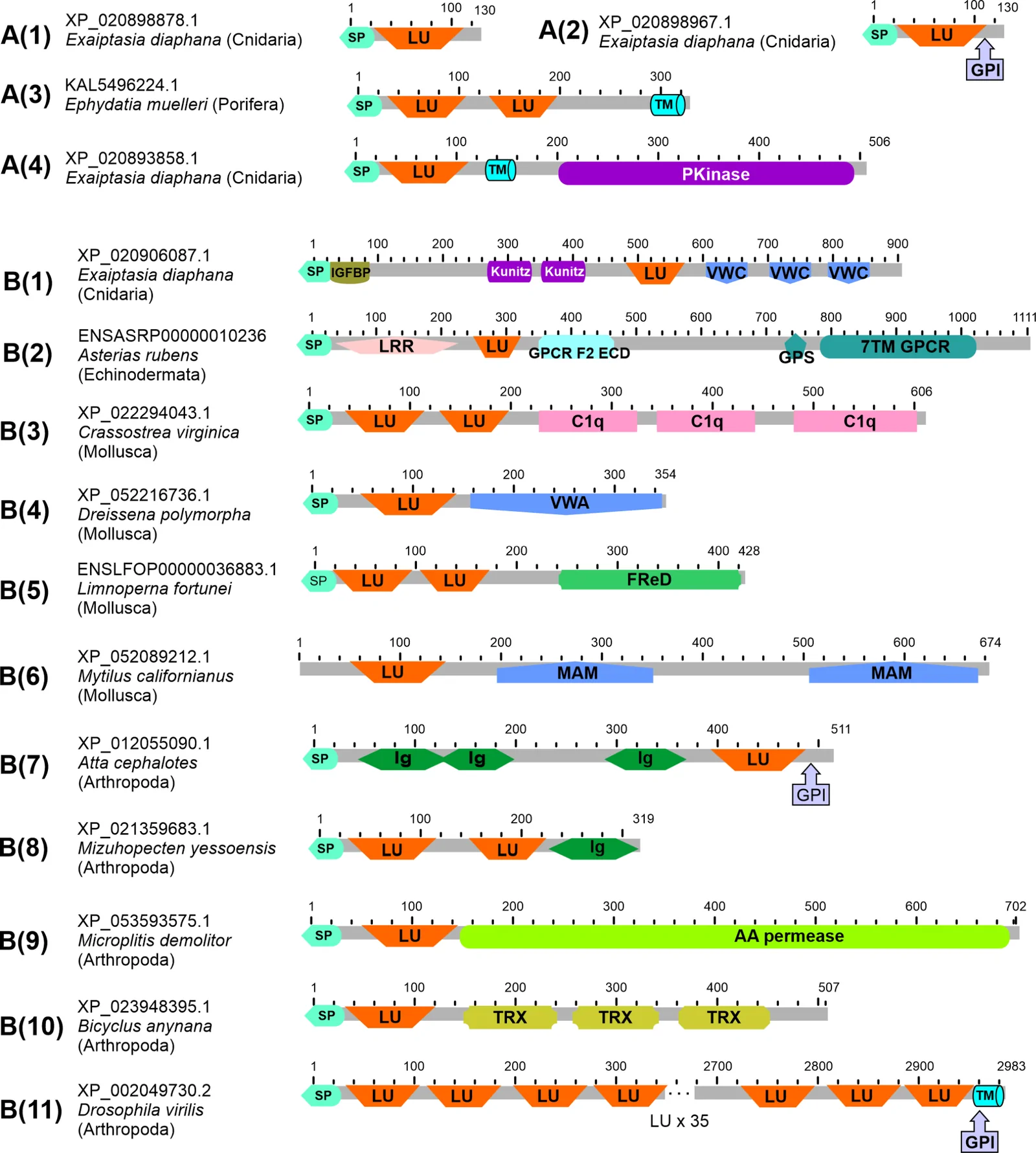
Domain organization of some invertebrate TFPs. Sequence accession numbers and the organism in whose genome the sequence was found are shown. (*A*) Examples of TFPs with the standard domain architecture. (*B*) Domain architectures not previously observed in TFPs. Domain legend: 7TM GPCR – the domain with 7TM helices (GPCR-like); AA premease – amino acid permease-like domain; C1q – subunit of the C1 enzyme complex; FreD – fibrinogen-like domain; GPI – GPI-anchor attachment site; GPS – autoproteolytic domain of adhesion GPCR; Ig – immunoglobulin-like domain; IGFBP – insulin/growth factor binding-like domain; Kunitz – Kunitz-type domain; LRR – leucine-rich repeat domain; LU – three-finger domain; MAM – MAM-type domain (meprin, A5); PKinase – protein kinase domain; SP – extracellular/membrane localization signal peptide; TM – transmembrane helix; TRX – thioredoxin-type domain; VWA and VWC – domains similar to the domains A and C of the von Willebrand factor

**Fig. 3:**
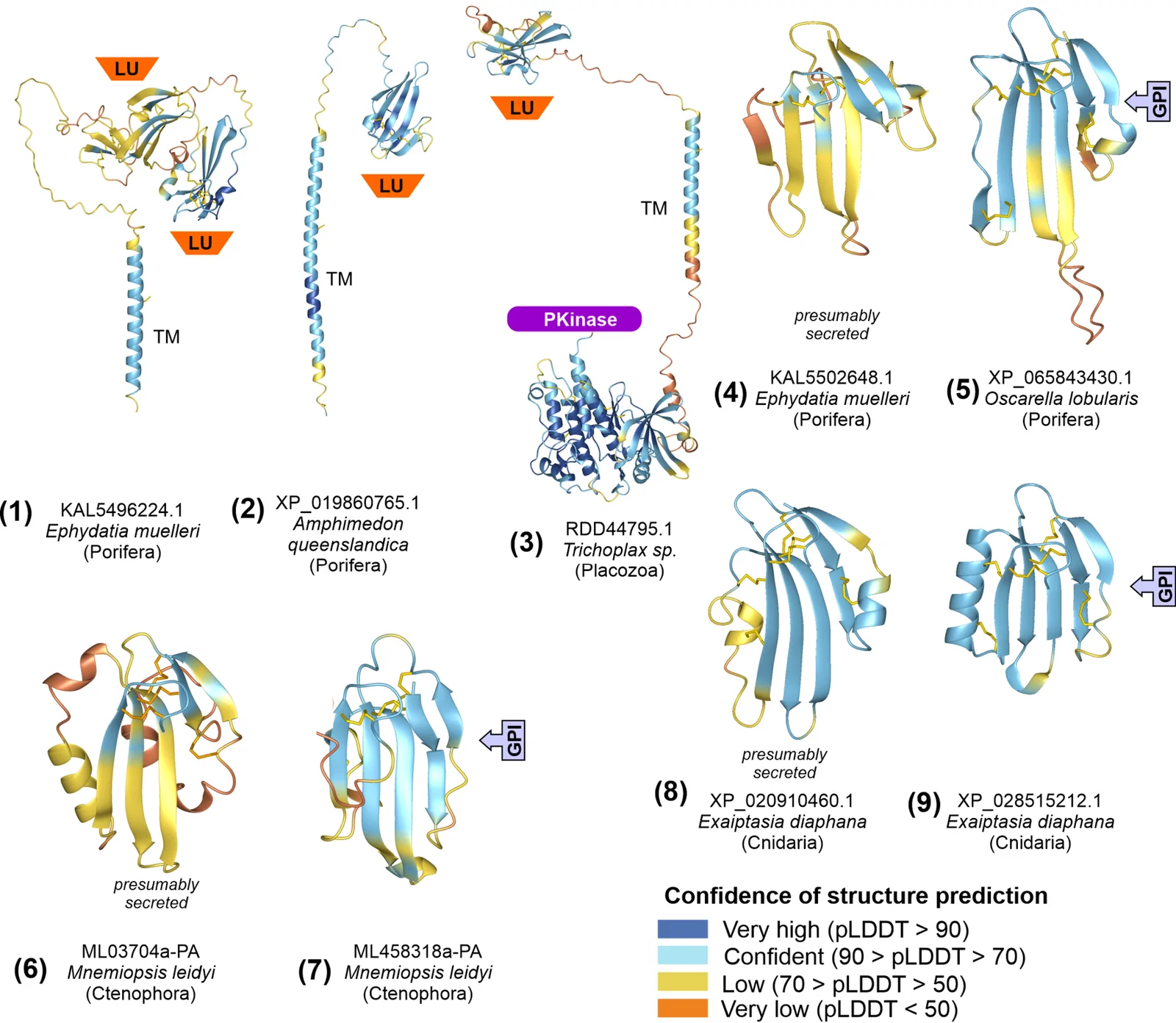
Spatial structures of the “classical” TFPs found in sponges, ctenophores, placozoans, and cnidarians, predicted from the sequence using the AlphaFold3 [25] or EsmFold tool [40]. The color code of prediction confidence (pLDDT) is shown. Domain designations are given in the legend to *Fig. 2*

The results of the analysis are interesting from the perspective of metazoan phylogeny. Thus, sponges, which possess no nervous system, muscles, differentiated tissues, or intercellular contacts and essentially are aggregates of cells [42], carry three-finger proteins with the same set of domains as that in higher animals. Ctenophores are an invertebrate phylum fundamentally distinct from other animals. Ctenophores are currently increasingly often assigned to a separate branching lineage of Metazoa (the “ctenophore-sister” hypothesis versus the previously accepted “sponge-sister” hypothesis [43]). In ctenophores, the nervous and muscular systems emerged and evolved independently of those in other metazoans. There also are fundamental structural differences: the nervous system of ctenophores lacks acetylcholine [44]. Meanwhile, the genes encoding both TGFβR-like TFPs and proteins harboring individual secreted or membrane-anchored LU domains were also identified in the genomes of ctenophores and sponges (*Fig. 3*). In mammals, TGFβR family receptors regulate tissue differentiation processes, which are fundamental to metazoans [6]. The fact of a widespread presence of these proteins was also alluded to earlier [45]. The presence of structurally diverse TFPs in sponges and ctenophores suggests that they may have possessed evolutionarily ancient functions, such as involvement in cell adhesion and recognition, as well as intercellular signaling. Meanwhile, it would appear that the secreted TFPs of phylogenetically early-branching animal phyla may hypothetically function as toxins or antimicrobial peptides for host defense, as well as act as repellents or attractants for members of the same or other species.

On the other hand, the placozoan *Trichoplax* is a phylogenetically distinct and highly reduced metazoan lacking a nervous and muscular system but containing a number of genes characteristic of much more complex animals [46]. The absence of diversity in the TFP genes pool in the *Trichoplax* genome may be testament of their reduction. Analysis of the genomes of other placozoans will shed light on this question.

**TFPs with a non-standard domain architecture**

An analysis of the molluscan and arthropod genomes revealed genes thought to encode GPCR receptors that contain an LU domain in their N-terminal extracellular region (*Figs. 2B* (2) and *4* (2)). Similar genes were first identified in the genome of the starfish *Asterias rubens* [7]; however, those genes were not found in the genomes of animals from other phyla, including mammals. Taking into account the significant evolutionary distance between these animal phyla (*Fig. 1*), it is fair to assume that the genes encoding TFPs with this architecture either have appeared independently or have been lost or modified in other phyla.

**Fig. 4:**
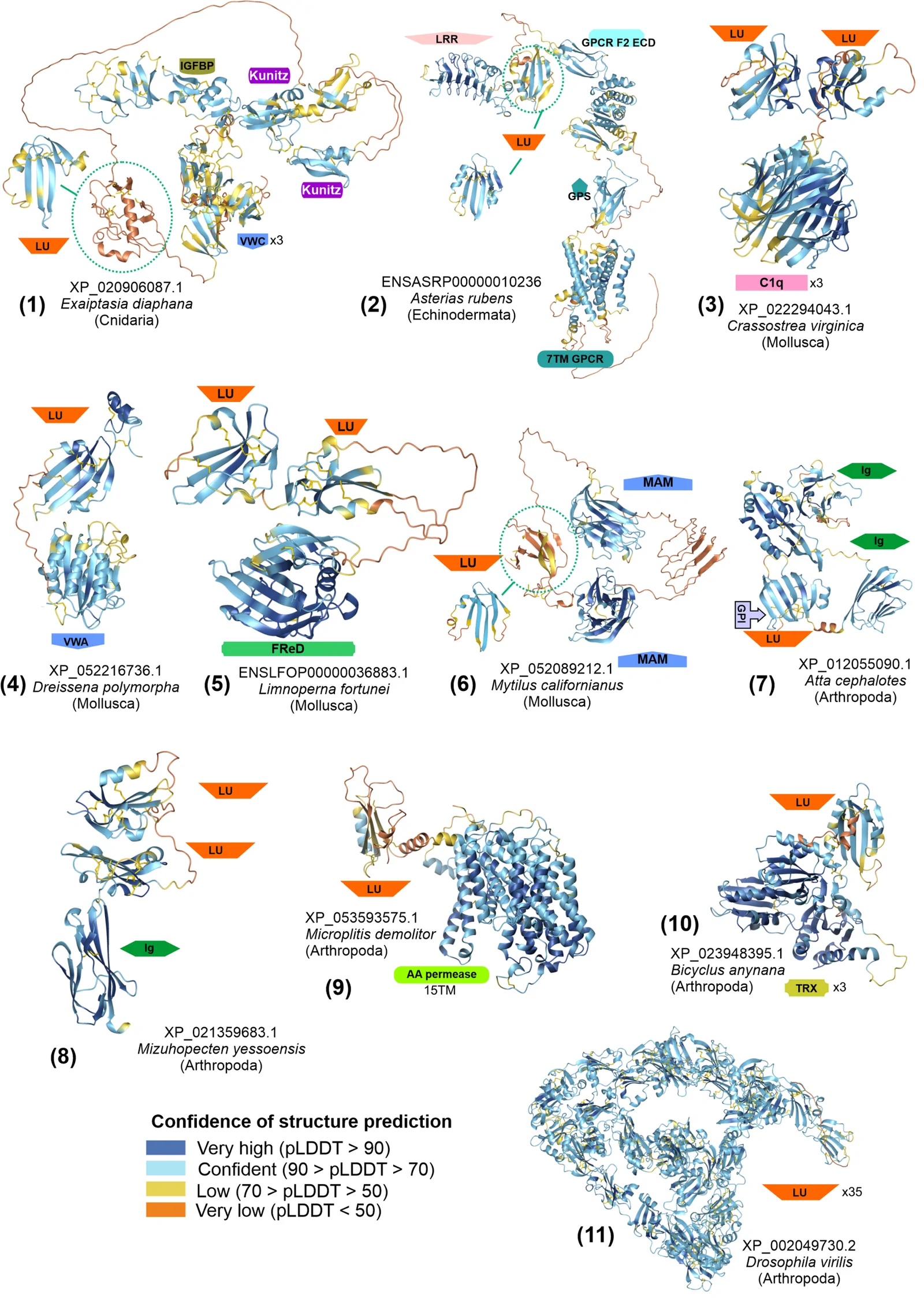
Spatial structures of TFPs with a non-standard domain architecture (*Fig. 2B*) predicted from the sequence. For proteins with low-quality prediction of the LU domain in the full-length protein (1, 2, and 6), the domain structures predicted separately are shown next to them

Along with proteins with a known domain architecture, we identified TFPs containing domain combinations that have not yet been described (*Figs. 2B* and *4*). Thus, a gene encoding the protein that carries Kunitz-type domains characteristic of serine protease inhibitors, together with the LU domain, was found in cnidarians (*Figs. 2B* (1) and *3* (1)). It apparently is a secreted protein, since it contains neither a GPI-anchor attachment site nor TM helices. Secreted Kunitz-type peptides are known to be widely represented in cnidarian venoms and, along with acting as protease inhibitors, also act as ion channel agonists and antagonists [47].

In mollusks, we identified the gene encoding the TFP that contains C1q domains characteristic of the subunit of the C1 complex involved in complement system activation in mammals (*Figs. 2B* (3) and *4* (3)), as well as the gene encoding the TFP with a domain similar to mammalian fibrinogen (*Figs. 2B* (5) and *4* (5)). Some TFP sequences from mollusks and arthropods contained, apart from LU domains, one to three immunoglobulin-like domains (*Figs. 2B* (7, 8) and *4* (7, 8)) or MAM-type domains (meprin, A5; *Figs. 2B* (6) and *4* (6)), which are found in the extracellular ligand-binding regions of many receptors involved in intercellular interactions.

Moreover, in arthropods, we identified genes encoding proteins that contain three thioredoxin-type domains, similar to the gene coding for human endoplasmic reticulum protein 44 (*Figs. 2B* (10) and *4* (10)), and the domain containing 15 TM helices, similar to amino acid permease (*Figs. 2B* (9) and *4* (9)). It is worth mentioning that TFPs with a non-standard architecture were found only in individual phyla and were not common across metazoans, suggesting that they have appeared independently.

**The close similarity between certain LU domains in basal Metazoa and humans**

We compared the LU-domain sequences of sponges, ctenophores, cnidarians, and placozoans to those of human TFPs. *Figure 5* shows the pairwise comparison matrix for the most similar sequences, while the full pairwise comparison matrix is presented in *Supplementary File 3* (*Supplementary Materials*). Many LU-domain pairs were found to have a relatively low similarity (the average similarity across the entire matrix is 49%; the minimal similarity mainly depending on conserved Cys residues is 28%), which is unsurprising considering the marked evolutionary divergence.

**Fig. 5:**
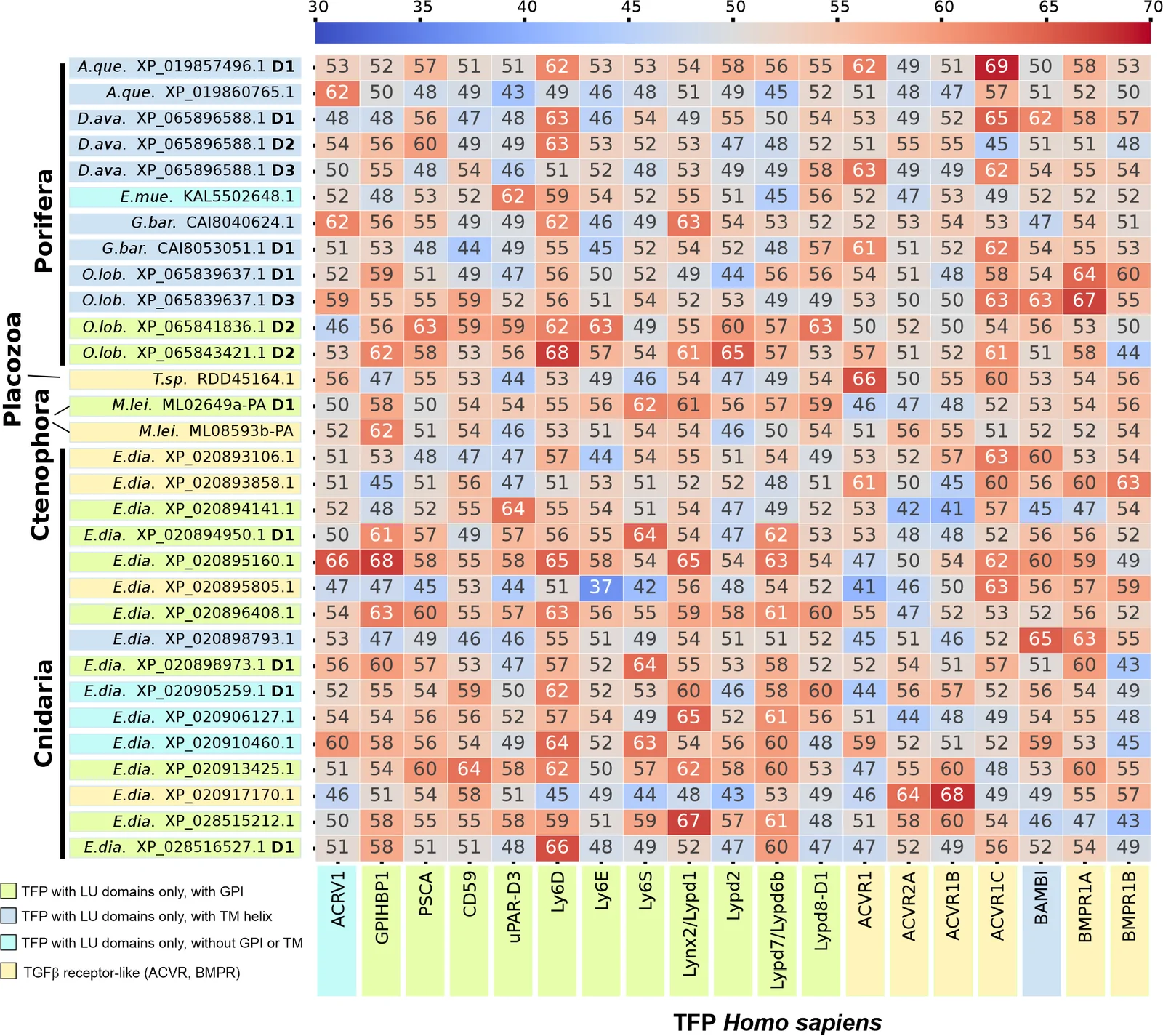
Heatmap illustrating the pairwise sequence similarity of LU domains from sponges, ctenophores, cnidarians, and placozoans with human LU domains. The LU domains with at least 62% similarity to at least one human domain are shown. The full pairwise comparison matrix is presented in *Supplementary File 3*. D1, D2, and D3 denote different domains of the same protein

The closest (up to 69%) similarity to TFPs of basal organisms was observed for human activin and BMP receptors, as well as the membrane protein BAMBI similar to them (*Fig. 5*). Genes of these proteins containing LU domains similar to those of human TFPs were previously encountered in starfish [7]. Activin receptors play a crucial role in cellular differentiation and ontogenesis [6]. These signaling cascades are presumably conserved and were essential back at the early stages of multicellularity evolution.

Proteins of basal organisms were also found to be highly similar to certain human TFPs containing isolated LU domains (*Fig. 5*). For example, close similarity (up to 68%) was observed between the human GPI-anchored proteins Ly6D and Lynx2 (Lypd1), which play a crucial role in lymphocyte differentiation [8] and CNS function [11], respectively. A protein homologous to human Lynx2 was previously characterized in echinoderms [24].

## CONCLUSIONS

It has been demonstrated that genes encoding three-finger proteins containing LU domains are found in all metazoans, including members of the most basal phyla (sponges, placozoans, and cnidarians), as well as in the phylogenetically distinct taxon Ctenophora. The absence of TFP genes in plants, fungi, and choanoflagellates is indication that the three-finger structural motif emerged during the evolution of protists, upon their transition to multicellularity. Although functional annotation of these proteins based solely on genomic data is challenging, comparative sequence analysis of basal metazoan proteins and the TFPs found in humans reveals conserved TFP subtypes and allows one to infer their function in the organism. TFPs have presumably been involved in essential biological processes from the earliest stages of the metazoan life. Although respective mRNAs in the transcriptomes of related species were detected, further proteomic research is needed in order to confirm the hypothesis that TFP proteins are synthesized in diverse organisms.

## Abbreviations

GPCR: G protein-coupled receptor
GPI: glycosylphosphatidylinositol
LU domain: three-finger (LU) domain
TGFβR: transforming growth factor receptor
RK: receptor kinase
TM: transmembrane
TFP: three-finger protein (in this study, any protein containing LU domains)
